# *SAP30BP* gene is associated with the susceptibility of rotator cuff tear: a case-control study based on Han Chinese population

**DOI:** 10.1186/s13018-020-01888-z

**Published:** 2020-08-26

**Authors:** Bin Tian, Xin Kang, Liang Zhang, Jiang Zheng, Zandong Zhao

**Affiliations:** Department of Sports Medicine, Honghui Hospital, Xi’an Jiaotong University Health Science Center, No.555, Youyi East Road, Xi’an, Shaanxi China

**Keywords:** *SAP30BP*, Single nucleotide polymorphism, Genetic susceptibility, Rotator cuff tear

## Abstract

**Background:**

Multiple studies have indicated that genetic components contribute significantly to the risk of rotator cuff tears. Previous studies have suggested that the *SAP30BP* gene may play an essential role in the development of rotator cuff tears. The aim of this study was to evaluate the potential association of the *SAP30BP* gene with the susceptibility to rotator cuff tears in a Han Chinese population.

**Methods:**

A total of 394 patients with rotator cuff tears and 998 healthy controls were included in the study. Twelve tag single nucleotide polymorphisms (SNPs) located in the region of the *SAP30BP* gene were selected for genotyping. Genetic association analyses were performed using *χ*^2^ tests for each SNP. Significant associations were searched in the GTEx database for their functional consequences.

**Results:**

SNP rs820218 was significantly associated with rotator cuff tears (*χ*^2^ = 9.49, *P* = 0.0021, OR [95% CI] = 0.67 [0.52–0.87]). In addition, SNP rs820218 was found to be significantly associated with the gene expression level of *SAP30BP* in whole blood (NES = 0.12, *P* = 1.00 × 10^−6^).

**Conclusion:**

Our study has shown that the genetic polymorphism of *SAP30BP* contributes to the risk of rotator cuff tears in Chinese Han people. Individuals with the *A* allele for SNP rs820218 were less susceptible to developing rotator cuff tears.

## Background

With the aging of society and the overuse injuries of shoulders occurring in sports as well as in jobs, the incidence of rotator cuff tears has increased from 5 to 39%, seriously harming human health and quality of life [1]. Recently, some genetic studies have shown that genetic factors might contribute to the development of rotator cuff tears [2, 3]. It has been reported that the prevalence rates of rotator cuff tears in first- and second-degree relatives of patients were significantly higher than that in the general population [4]. Moreover, researchers have also found that rotator cuff tears in siblings are more likely to progress over a period of 5 years [5]. In addition, a related study reported that the second- and third-degree relatives of younger rotator cuff tear patients (younger than 40 years old) have a significantly increased relative risk for developing tears than those of older patients [6]. However, the understanding of the etiology and pathogenesis of rotator cuff tears remains unclear [7]. Given that genetics has been investigated as a factor involved in the pathogenesis of rotator cuff pathology [1], it is urgent that the susceptibility gene to rotator cuff tears and the molecular mechanism of this disease are clearly identified.

Previously, Maffulli et al. have focused their attention on the study of genetic susceptibility of rotator cuff tears [1, 7–9], and many susceptibility genes associated with rotator cuff tears have been identified through linkage analyses in candidate gene association studies and genome-wide association studies (GWAS) [10, 11]. Among them, a recently published study documented the results of the GWASs on rotator cuff tears conducted to date and identified significant evidence of an association of rs820218 in the SAP30-binding protein (*SAP30BP*) gene with rotator cuff tears in the American population [11]. It has been reported that *SAP30BP* inhibits transcription and induces apoptosis by interacting with the SAP30-associated mSin3 complex [12]. By assessing the edges of torn supraspinatus rotator cuff tendons collected during surgery on patients with rotator cuff tears, the authors have found excessive apoptosis in rotator cuff tear patients, and this was a primary cause of tendinopathy [13]. Furthermore, researchers have also found that upregulated p53 may induce apoptosis in patients with torn supraspinatus tendons compared with controls using immunohistochemistry [14]. Hence, it is reasonable to suppose that the *SAP30BP* gene may be involved in the occurrence and development of rotator cuff tears by inducing apoptosis in tendons.

However, the most recent GWAS did not show a significant association between the *SAP30BP* gene and rotator cuff tears [15]. Given that different ethnic populations may exhibit genetic heterogeneity related to rotator cuff tears, replications of the study using more samples from different populations are needed to confirm these results. Currently, no information is available on the Han Chinese population regarding the *SAP30BP* gene and rotator cuff tears. Therefore, in the present study, we conducted a case-control association study to evaluate the relationship between the *SAP30BP* gene and rotator cuff tears in a Han Chinese population.

## Methods

### Study subjects

In this study, we recruited 394 patients with rotator cuff tears and 998 controls from Honghui Hospital of Xi’an Jiaotong University from May 2016 to April 2019. All patients who had rotator cuff tears were under 70 years of age. Patients with rheumatism, diabetes, previous shoulder surgery, traumatic rotator cuff tears, and infection in the shoulder joint were excluded. The patient group and the control group were age-matched so that the maximum age difference was not more than 2 years. The controls were patients who underwent trauma treatment at the same hospital, but they did not have clinical symptoms associated with shoulder pain or other rotator cuff disorders. All samples were examined by imaging of the bilateral rotator cuff (MRI or ultrasonography). The rotator cuff tendons were intact in all subjects in the control group. Demographic information on all study subjects was collected using interviews. Informed consent forms were signed by all subjects. This study was reviewed and approved by the ethics committee of Honghui Hospital of Xi’an Jiaotong University.

### SNP selection and genotyping

SNPs located within the region of the *SAP30BP* gene with minor allele frequencies (MAF) greater than 0.05 were chosen from the Chinese Han Beijing (CHB) 1000 genome dataset. Then, tag SNPs with *r*^2^ ≥ 0.7 were selected from the selected SNP set. A total of 12 tag SNPs were included for genotyping (Supplemental Table S1). In accordance with the manufacturer’s protocol (Genomic DNA kit, Axygen Scientific, Inc., CA, USA), we extracted genomic DNA from peripheral blood leukocytes. A high-throughput Sequenom MassARRAY platform (Sequenom, San Diego, CA, USA) was utilized for SNP genotyping. Briefly, the signals from the platform were automatically analyzed using Sequenom Typer 4.0 software, and genotype data were generated from the processed results [16]. To estimate the genotyping quality, 5% of the random samples were repeated for genotyping [17]. The concordance rate was 100%, so the quality of the genotyping data was confirmed.

### Statistical analyses

Hardy-Weinberg equilibrium (HWE) tests were conducted within the control group for each SNP. Genetic association analyses were performed for each SNP by using *χ*^2^ tests and Plink [18]. The effect size of the association between the minor allele of each SNP and the disease status of the rotator cuff tear was presented as an odds ratio. Bonferroni corrections were applied for multiple comparisons. The statistical significance threshold for the *P* values was 0.05/12≈0.004 for single marker-based association analyses. A linkage disequilibrium (LD) plot was made using Haploview [19] to virtualize the LD structure of the 12 selected SNPs in our samples. To further investigate the functional consequences of significant SNPs, we examined these SNPs in the GTEx database [20] to establish the link between genotypes of these SNPs and gene expression levels in multiple human tissues. Significant expression quantitative trait loci (eQTLs) from various human tissues were recorded.

## Results

**Fig. 1:**
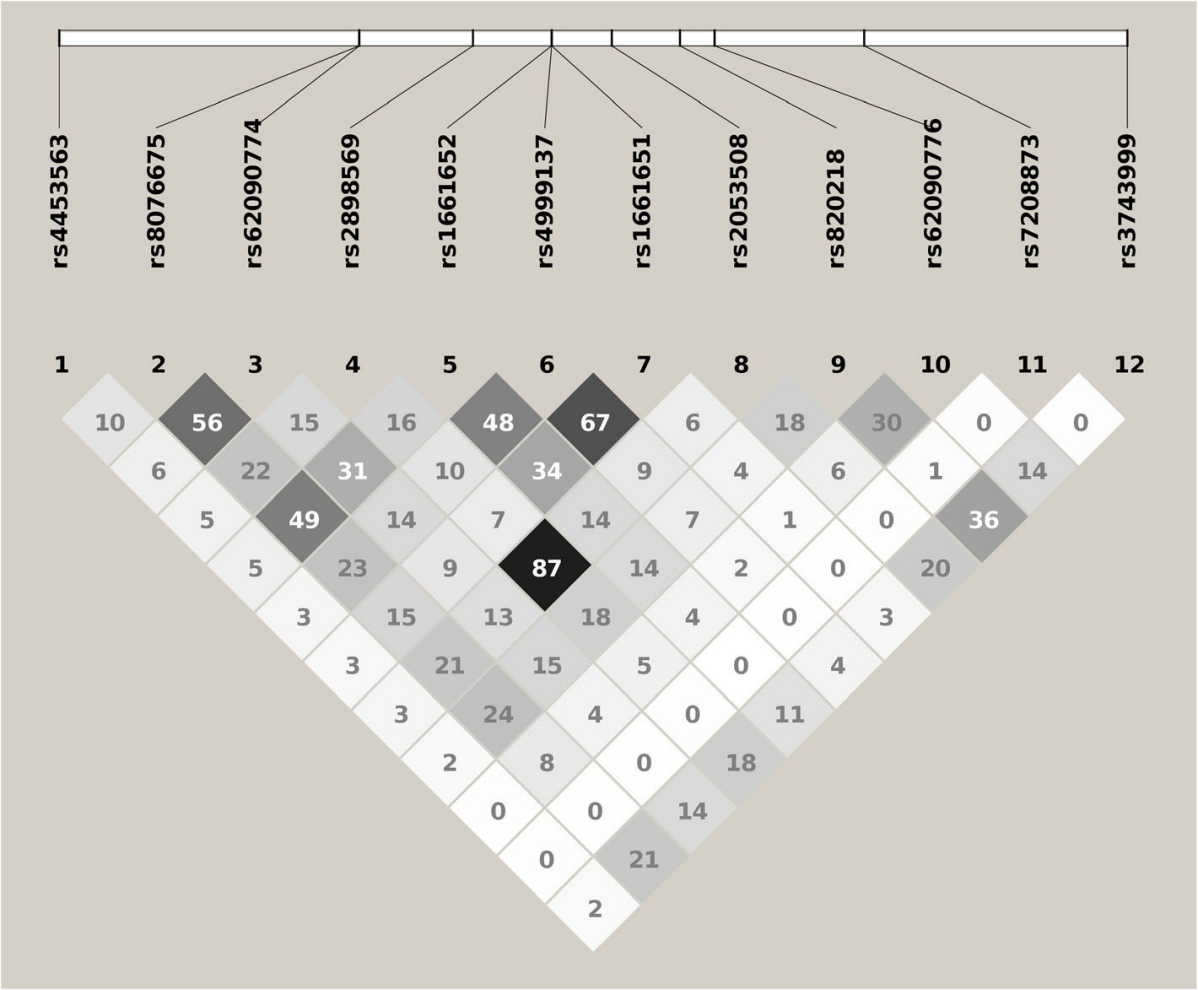
Linkage disequilibrium plot of the 12 genotyped SNPs in study subjects. Values of *r*^2^ are indicated in each cell and are utilized by different gray shading. The darker the gray color, the greater the value

A total of 394 patients with rotator cuff tears and 998 healthy controls were included in the study (Table 1). No significant differences were observed in terms of the patient’s age, sex, the presence of other tendinopathies, smoking habits, drinking habits, work with repeated and sustained arm abduction or sports with shoulder involvement between the patients and controls. SNP rs820218 was significantly associated with rotator cuff tears (*χ*^2^ = 9.49, *P* = 0.0021, OR [95% CI] = 0.67 [0.52–0.87], Table 2). The MAF of this SNP was significantly lower in the patients than in the controls (0.11 vs. 0.15), and the study subjects with its minor allele, *A* allele, had a lower chance of having a rotator cuff tear. No other SNPs were found to be associated with rotator cuff tears. The LD plot showed that SNP rs820218 was modest to moderate in LD with the other genotyped SNPs (Fig. 1). We explored the SNP rs820218 in the GTEx database and identified that this SNP was significantly associated with the expression levels of five genes in various human tissues (Table 3). SNP rs820218 was found to be significantly associated with the expression level of the *SAP30BP* gene in whole blood. In addition to *SAP30BP*, SNP rs820218 was also identified as a significant eQTL hit for four other genes, including *RECQL5*, *SMIM6*, *ITGB4*, and *SMIM5,* in multiple human tissues.

**Table 1:** Demographic and clinical characteristic information on the study subjects

	Patients (*N* = 394)	Controls (*N* = 998)	Statistics	*P* value
Age, mean±sd	53.7 ± 6.6	54.1 ± 6.5	*T* = −1.02	0.31
Gender (%)				
Male	181 (46)	456 (46)		
Female	213 (54)	542 (54)	χ^2^ = 0.0006	0.98
Other tendinopathies (%)				
Yes	21 (5)	52 (5)		
No	373 (95)	946 (95)	*χ*^2^ = 6.66 × 10^−31^	1.00
Smoke (%)				
Yes	77 (20)	193 (19)		
No	317 (80)	805 (81)	*χ*^2^ = 0.0001	0.99
Drink alcohol (%)				
Yes	87 (22)	224 (22)		
No	307 (78)	774 (78)	*χ*^2^ = 0.0057	0.94
Work with repeated and sustained arm abduction (%)				
Yes	161 (41)	401 (40)		
No	233 (59)	597 (60)	*χ*^2^ = 0.0300	0.86
Perform sports with shoulder involvement (%)				
Yes	40 (10)	107 (11)		
No	354 (90)	891 (89)	*χ*^2^ = 0.0460	0.83

**Table 2:** Results of single marker-based genetic association analyses

CHR	SNP	POS	A1	F_A	F_U	A2	χ^2^	*P*	OR[95% CI]
17	rs4453563	75674697	T	0.28	0.27	G	0.05	0.82	1.02[0.85–1.23]
17	rs8076675	75682774	T	0.16	0.16	C	0.10	0.75	1.04[0.83–1.30]
17	rs62090774	75682779	C	0.12	0.11	T	0.07	0.79	1.04[0.80–1.34]
17	rs2898569	75685840	T	0.41	0.41	A	0.01	0.91	1.01[0.85–1.19]
17	rs1661652	75687957	A	0.25	0.25	T	0.01	0.91	0.99[0.82–1.20]
17	rs4999137	75687959	A	0.38	0.38	T	0.03	0.86	0.99[0.83–1.17]
17	rs1661651	75687961	A	0.31	0.31	T	0.01	0.91	0.99[0.83–1.18]
17	rs2053508	75689579	G	0.43	0.42	A	0.07	0.79	1.02[0.87–1.21]
**17**	**rs820218**	**75691415**	**A**	**0.11**	**0.15**	**G**	**9.49**	**0.0021**	**0.67[0.52–0.87]**
17	rs62090776	75692348	T	0.07	0.06	G	0.47	0.50	1.12[0.80–1.58]
17	rs7208873	75696377	C	0.26	0.25	A	0.05	0.82	1.02[0.85–1.24]
17	rs3743999	75703466	C	0.16	0.15	G	0.13	0.71	1.04 [0.83–1.31]

*CHR* chromosome, *SN*P single nucleotide polymorphism, *POS* position; *A1/A2*: minor/major allele; *F*_*A/F*_*U*: minor allele frequency of the patients/controls.

A significant result is indicated in bold font. The statistical significance threshold of the *P* values was 0.05/12 ≈ 0.004.

**Table 3:** Significant eQTL signals for SNP rs820218 from multiple human tissues based on the GTEx data

GENE	SNP	*P*-Value	Ref	Alt	NES	Tissue
*RECQL5*	rs820218	5.60 × 10^-13^	G	A	0.27	Artery—Tibial
*RECQL5*	rs820218	1.70 × 10^-8^	G	A	-0.29	Testis
*RECQL5*	rs820218	1.80 × 10^-7^	G	A	0.17	Muscle—Skeletal
*SMIM6*	rs820218	2.10 × 10^-7^	G	A	-0.40	Adipose—Visceral (omentum)
*RECQL5*	rs820218	3.60 × 10^-7^	G	A	-0.24	Pituitary
*RECQL5*	rs820218	4.40 × 10^-7^	G	A	0.20	Adipose—Subcutaneous
*SAP30BP*	rs820218	1.00 × 10^-6^	G	A	0.12	Whole Blood
*RECQL5*	rs820218	1.60 × 10^-6^	G	A	0.17	Esophagus—Muscularis
*ITGB4*	rs820218	1.80 × 10^-6^	G	A	-0.14	Skin—Sun Exposed (Lower leg)
*ITGB4*	rs820218	4.40 × 10^-6^	G	A	-0.29	Pancreas
*SMIM5*	rs820218	4.50 × 10^-6^	G	A	-0.28	Adipose—Visceral (Omentum)
*SMIM5*	rs820218	8.10 × 10^-6^	G	A	-0.17	Heart—left ventricle

*SNP* single nucleotide polymorphism, *Ref* Reference allele, *Alt* alternative allele, *NES* normalized effect size

## Discussion

Our study showed that a genetic polymorphism located within the region of the *SAP30BP* gene was significantly associated with the disease status of rotator cuff tears. To the best of our knowledge, this significant hit was the first to be observed for rotator cuff tears in the Chinese Han population. The *A* allele of SNP rs820218 had a protective effect for rotator cuff tears, and this finding was in accordance with that in the recent GWAS conducted in European Americans, despite the reported effect size of SNP rs820218 in the GWAS being larger than that in the present study (OR = 0.40 vs OR = 0.67) [11]. This difference may be at least partly explained by the difference in allele frequency of the *A* allele for rs820218 between the Chinese Han population and the European American population. The *A* allele frequency of SNP rs820218 was much larger in European Americans than in Chinese Han populations. Our study can be considered a replicate study of the recent GWAS conducted in European Americans. However, if we consider that this previous GWAS had a very limited number of rotator cuff tear cases and lacked a built-in replication set, our candidate gene-based study conducted in a population different from the European American population is very valuable. The significant results observed in our study can considerably strengthen the credibility of the significant hit reported previously.

*SAP30BP* is located on chromosome 17q25.1 and encodes a transcriptional regulator protein. Increased levels of *SAP30BP* have been demonstrated to be present in synovial cells in rheumatoid arthritis (RA) patients, which indicates that *SAP30BP* may promote the death of related cells or some cytokines in patients with RA, preventing the prompt activation of certain immune pathways [21]. The immune system also plays an important role in the process of rotator cuff tears [22]. The inhibitor TGF-β1 can improve muscle outcomes following rotator cuff tears [23]. More importantly, TGF-β1 can induce the development of the SAP30-like protein [24]. These results suggest that the *SAP30BP* gene may play an essential role in the development of rotator cuff tears. Nevertheless, additional studies using animal models are needed to identify the underlying mechanism of rotator cuff tears and their relationship with *SAP30BP.*

SNP rs820218 is an intronic variant and cannot have a functional consequence on the changes in the protein sequence and structure of *SAP30BP*. Our bioinformatics analyses showed that SNP rs820218 was significantly associated with the gene expression level of *SAP30BP* in human whole blood. This finding indicated that SNP rs820218 might be a functional variant that has a true effect on the targeted disease. Nevertheless, we need to interpret our findings from the GTEx data with caution, as the data were obtained mainly from healthy individuals. Additional eQTL tests and analyses conducted in rotator cuff tear patients may strengthen the evidence. Interestingly, the gene expression levels of several genes neighboring *SAP30BP* were also significantly associated with the genotypes of SNP rs820218. However, since there is no biological evidence that connects these genes with bone metabolism or bone-related diseases, SNP rs820218 should not be functionally mapped to these genes.

With the continuous development of sequencing technology and its rapid decline in cost, more and more susceptibility variants of complex diseases have been reported, such as schizophrenia [25–27]. Because it is difficult to draw reliable conclusions only based on SNP analyses [28–32], our study has several limitations. We did not assess population stratification in our sample, which is considered to be the most important confounder for genetic association mapping. As this study was an association mapping study of candidate genes, only a few SNPs were genotyped, and it would be difficult for us to implement common statistical procedures (such as principal component analysis or genomic control) to account for this confounder. Nevertheless, in the sample recruitment process, we restricted our study subjects to be local people without a history of immigration within three generations. We believe that this procedure at least partly controlled for the potential population stratifications by reducing the genetic heterogeneity of the study subjects. In addition, in this study, we only selected SNPs located within the region of the *SAP30BP* gene. Potential functional SNPs located in the ±20 kb region of the *SAP30BP* gene were not considered in the present study. Therefore, the present study might have failed to cover some important genetic regions with high functional significance. Several recent studies have indicated that rare and low-frequency variants might play an important role in the pathology of complex disorders [33, 34]. Sequencing-based studies on genetic regions and the functional significance of *SAP30BP* are needed in the future to identify the genetic architecture of rotator cuff tears.

In summary, our study has shown that the genetic polymorphism of *SAP30BP* contributes to the risk of rotator cuff tears in Chinese Han populations. Individuals with *A* allele for SNP rs820218 are less susceptible to developing rotator cuff tears.

## Supplementary information

**Additional file 1: Table S1**. Basic information of the selected SNPs.

## Data Availability

Please contact the authors for reasonable requests.

## Abbreviations

GWAS: Genome-wide association study
*SAP30BP*: SAP30-binding protein
CHB: Chinese Han Beijing
HWE: Hardy-Weinberg equilibrium
LD: Linkage disequilibrium
eQTL: Expression quantitative trait loci
MAF: Minor allele frequency
ORs: Odds ratios
SNP: Single nucleotide polymorphism
NES: Normalized effect size

## References

[1] Longo UG, Berton A, Papapietro N, Maffulli N, Denaro V (2012). Epidemiology, genetics and biological factors of rotator cuff tears. Med Sport Sci.

[2] Orth T, Pare J, Froehlich JE (2017). current concepts on the genetic factors in rotator cuff pathology and future implications for sports physical therapists. Int J Sports Phys Ther.

[3] Motta Gda R, Amaral MV, Rezende E, Pitta R, Vieira TC, Duarte ME (2014). Evidence of genetic variations associated with rotator cuff disease. J Shoulder Elb Surg.

[4] Harvie P, Ostlere SJ, Teh J, McNally EG, Clipsham K, Burston BJ (2004). Genetic influences in the aetiology of tears of the rotator cuff. Sibling risk of a full-thickness tear. J Bone Joint Surg (Br).

[5] Gwilym SE, Watkins B, Cooper CD, Harvie P, Auplish S, Pollard TC (2009). Genetic influences in the progression of tears of the rotator cuff. J Bone Joint Surg (Br).

[6] Tashjian RZ, Farnham JM, Albright FS, Teerlink CC, Cannon-Albright LA (2009). Evidence for an inherited predisposition contributing to the risk for rotator cuff disease. J Bone Joint Surg Am.

[7] Longo UG, Margiotti K, Petrillo S, Rizzello G, Fusilli C, Maffulli N (2018). Genetics of rotator cuff tears: no association of col5a1 gene in a case-control study. BMC Med Genet.

[8] Maffulli N, Longo UG, Berton A, Mattia L, Denaro V (2011). Biological factors in the pathogenesis of rotator cuff tears. Sports Med Arthrosc Rev.

[9] Oliva F, Barisani D, Grasso A, Maffulli N (2011). Gene expression analysis in calcific tendinopathy of the rotator cuff. Eur Cell Mater.

[10] Assunção JH, Godoy-Santos AL, Dos Santos MCLG, Malavolta EA, Gracitelli MEC, Ferreira Neto AA (2017). Matrix metalloproteases 1 and 3 promoter gene polymorphism is associated with rotator cuff tear. Clin Orthop Relat Res.

[11] Tashjian RZ, Granger EK, Farnham JM, Cannon-Albright LA, Teerlink CC (2016). Genome-wide association study for rotator cuff tears identifies two significant single-nucleotide polymorphisms. J Shoulder Elb Surg.

[12] Li JF, Liu LD, Ma SH, Che YC, Wang LC, Dong CH (2004). HTRP—an immediate-early gene product induced by HSV1 infection in human embryo fibroblasts, is involved in cellular co-repressors. J Biochem.

[13] Yuan J, Murrell GA, Wei AQ, Wang MX (2002). Apoptosis in rotator cuff tendonopathy. J Orthop Res.

[14] Lundgreen K, Lian OB, Engebretsen L, Scott A (2011). Tenocyte apoptosis in the torn rotator cuff: a primary or secondary pathological event?. Br J Sports Med.

[15] Roos TR, Roos AK, Avins AL, Ahmed MA, Kleimeyer JP, Fredericson M (2017). Genome-wide association study identifies a locus associated with rotator cuff injury. PLoS One.

[16] Guan F, Zhang C, Wei S, Zhang H, Gong X, Feng J (2012). Association of PDE4B polymorphisms and schizophrenia in Northwestern Han Chinese. Hum Genet.

[17] Guan F, Zhang B, Yan T, Li L, Liu F, Li T (2014). MIR137 gene and target gene CACNA1C of miR-137 contribute to schizophrenia susceptibility in Han Chinese. Schizophr Res.

[18] Chang CC, Chow CC, Tellier LC, Vattikuti S, Purcell SM, Lee JJ (2015). Second-generation PLINK: rising to the challenge of larger and richer datasets. Gigascience..

[19] Barrett JC, Fry B, Maller J, Daly MJ (2005). Haploview: analysis and visualization of LD and haplotype maps. Bioinformatics..

[20] Consortium GT (2013). The Genotype-Tissue Expression (GTEx) project. Nat Genet.

[21] Wang H, Guo J, Jiang J, Wu W, Chang X, Zhou H (2017). New genes associated with rheumatoid arthritis identified by gene expression profiling. Int J Immunogenet.

[22] Bedi A, Maak T, Walsh C, Rodeo SA, Grande D, Dines DM (2012). Cytokines in rotator cuff degeneration and repair. J Shoulder Elb Surg.

[23] Davies MR, Liu X, Lee L, Laron D, Ning AY, Kim HT (2016). TGF-beta Small Molecule Inhibitor SB431542 Reduces Rotator Cuff Muscle Fibrosis and Fatty Infiltration By Promoting Fibro/Adipogenic Progenitor Apoptosis. PLoS One.

[24] Lindfors K, Viiri KM, Niittynen M, Heinonen TY, Mäki M, Kainulainen H (2003). TGF-β induces the expression of SAP30L, a novel nuclear protein. BMC Genomics.

[25] Zhang T, Zhu L, Ni T, Liu D, Chen G, Yan Z (2018). Voltage-gated calcium channel activity and complex related genes and schizophrenia: A systematic investigation based on Han Chinese population. J Psychiatr Res.

[26] Han W, Zhang T, Ni T, Zhu L, Liu D, Chen G (2019). Relationship of common variants in CHRNA5 with early-onset schizophrenia and executive function. Schizophr Res.

[27] Guan F, Ni T, Han W, Lin H, Zhang B, Chen G (2020). Evaluation of the relationships of the WBP1L gene with schizophrenia and the general psychopathology scale based on a case–control study. Am J Med Genet B Neuropsychiatr Genet.

[28] Zhu L, Li J, Dong N, Guan F, Liu Y, Ma D (2016). mRNA changes in nucleus accumbens related to methamphetamine addiction in mice. Sci Rep.

[29] Sun H, Luo C, Chen X, Tao L (2017). Assessment of cognitive dysfunction in traumatic brain injury patients: a review. Forensic Sci Res.

[30] Zhang Z, Gong Q, Feng X, Zhang D, Quan L (2017). Astrocytic clasmatodendrosis in the cerebral cortex of methamphetamine abusers. Forensic Sci Res.

[31] Li J, Zhu L, Guan F, Yan Z, Liu D, Han W (2018). Relationship between schizophrenia and changes in the expression of the long non-coding RNAs Meg3, Miat, Neat1 and Neat2. J Psychiatr Res.

[32] Guan F, Zhang T, Han W, Zhu L, Ni T, Lin H (2020). Relationship of SNAP25 variants with schizophrenia and antipsychotic-induced weight change in large-scale schizophrenia patients. Schizophr Res.

[33] Sule G, Campeau PM, Zhang VW, Nagamani SC, Dawson BC, Grover M (2013). Next-generation sequencing for disorders of low and high bone mineral density. Osteoporos Int.

[34] Umair M, Alhaddad B, Rafique A, Jan A, Haack TB, Graf E (2017). Exome sequencing reveals a novel homozygous splice site variant in the WNT1 gene underlying osteogenesis imperfecta type 3. Pediatr Res.

