# Supplementary material for: SAP30BP gene is associated with the susceptibility of rotator cuff tear: a case-control study based on Han Chinese population
Source: J Orthop Surg Res. 2020 Aug 26;15:356. doi: 10.1186/s13018-020-01888-z (PMC7449091; doi:10.1186/s13018-020-01888-z)
Supplement: Supplementary file 1 — Additional file 1: Table S1. Basic information of the selected SNPs. [file 13018_2020_1888_MOESM1_ESM.docx]

Supplemental Table S1. Basic information of the selected SNPs.

| CHR | POS | SNP | FUNC | A1 | A2 | MAF | HWE |
| --- | --- | --- | --- | --- | --- | --- | --- |
| 17 | 75674697 | rs4453563 | intron | T | G | 0.27 | 0.52 |
| 17 | 75682774 | rs8076675 | intron | T | C | 0.16 | 0.48 |
| 17 | 75682779 | rs62090774 | intron | C | T | 0.11 | 0.43 |
| 17 | 75685840 | rs2898569 | intron | T | A | 0.41 | 0.74 |
| 17 | 75687957 | rs1661652 | intron | A | T | 0.25 | 0.50 |
| 17 | 75687959 | rs4999137 | intron | A | T | 0.38 | 0.79 |
| 17 | 75687961 | rs1661651 | intron | A | T | 0.31 | 0.71 |
| 17 | 75689579 | rs2053508 | intron | G | A | 0.42 | 0.52 |
| 17 | 75691415 | rs820218 | intron | A | G | 0.14 | 0.22 |
| 17 | 75692348 | rs62090776 | intron | T | G | 0.06 | 0.38 |
| 17 | 75696377 | rs7208873 | intron | C | A | 0.25 | 0.56 |
| 17 | 75703466 | rs3743999 | intron | C | G | 0.15 | 0.81 |

CHR: chromosome; POS: position; FUNC: function; A1: minor allele; A2: major allele; MAF: minor allele frequency; HWE: *P* values of Hardy-Weinberg equilibrium tests conducted in controls.
